# Maternal stress and postnatal hospitalization of the baby

**DOI:** 10.1192/j.eurpsy.2023.1514

**Published:** 2023-07-19

**Authors:** K. Chiha, K. Khemakhem, C. Regaieg, D. Ben Touhemi, H. Ayadi, N. Hmida, A. Gargouri, Y. Moalla

**Affiliations:** 1 Child Psychiatry; 2 NeonatologyDepartment, Hedi Chaker University Hospital, Sfax, Tunisia

## Abstract

**Introduction:**

Postnatal hospitalisation is an extremely traumatic event for both mother and baby. Such a situation reflects a psychological dysfunction with a risk of developing a post-traumatic stress disorder.

**Objectives:**

To study the level of stress in mothers of babies hospitalised during the postnatal period in the neonatal unit and to identify the risk factors associated with the persistence of high levels of stress 3 months after discharge.

**Methods:**

This was a longitudinal, descriptive and analytical study conducted between April and September 2021. The sample consisted of mothers of babies hospitalized in the neonatology department of Sfax-Tunisia for a period ranging from 5 to 15 days. Socio-demographic data were collected using a pre-designed form. The level of stress was assessed using the 22-item “Impact of Event Scale-Revised” (IES-R), validated in Arabic.

**Results:**

The sample consisted of 86 mothers with a mean age of 32.17 years.

Severe stress symptoms were found in 77.90% of the mothers during their babies’ hospitalisation. They persisted in 8.90% of the young mothers 3 months after discharge from hospital.

Certain factors were significantly associated with the persistence of a high level of stress in mothers 3 months after the discharge of their babies, such as the occurrence of postpartum complications (p=0.012), the absence of visits to the baby’s intensive care unit (p=0.047) and a living environment with a single parent (p=0.034).

**Conclusions:**

The present study shows that the level of stress is quite high among mothers of babies hospitalised in neonatology during the postnatal period and that this symptomatology can last for months after discharge.

Prevention and reduction of stress induced by postnatal hospitalisation through parental guidance and psychological support for mothers strengthens interactions and the mother-baby bond.

**Disclosure of Interest:**

None Declared

